# Overcoming Dietary Assessment Challenges in Low-Income Countries: Technological Solutions Proposed by the International Dietary Data Expansion (INDDEX) Project

**DOI:** 10.3390/nu9030289

**Published:** 2017-03-16

**Authors:** Jennifer C. Coates, Brooke A. Colaiezzi, Winnie Bell, U. Ruth Charrondiere, Catherine Leclercq

**Affiliations:** 1 Friedman School of Nutrition Science and Policy, Tufts University, Boston, MA 02111, USA; brooke.colaiezzi@tufts.edu (B.A.C.); winnie.bell@tufts.edu (W.B.); 2 Food and Agriculture Organization of the United Nations, Roma 00153, Italy; ruth.charrondiere@fao.org (U.R.C.); catherine.leclercq@fao.org (C.L.)

**Keywords:** individual-level dietary assessment, dietary intake, automated assessment, technology, innovation, low-income countries, developing countries, food composition

## Abstract

An increasing number of low-income countries (LICs) exhibit high rates of malnutrition coincident with rising rates of overweight and obesity. Individual-level dietary data are needed to inform effective responses, yet dietary data from large-scale surveys conducted in LICs remain extremely limited. This discussion paper first seeks to highlight the barriers to collection and use of individual-level dietary data in LICs. Second, it introduces readers to new technological developments and research initiatives to remedy this situation, led by the International Dietary Data Expansion (INDDEX) Project. Constraints to conducting large-scale dietary assessments include significant costs, time burden, technical complexity, and limited investment in dietary research infrastructure, including the necessary tools and databases required to collect individual-level dietary data in large surveys. To address existing bottlenecks, the INDDEX Project is developing a dietary assessment platform for LICs, called INDDEX24, consisting of a mobile application integrated with a web database application, which is expected to facilitate seamless data collection and processing. These tools will be subject to rigorous testing including feasibility, validation, and cost studies. To scale up dietary data collection and use in LICs, the INDDEX Project will also invest in food composition databases, an individual-level dietary data dissemination platform, and capacity development activities. Although the INDDEX Project activities are expected to improve the ability of researchers and policymakers in low-income countries to collect, process, and use dietary data, the global nutrition community is urged to commit further significant investments in order to adequately address the range and scope of challenges described in this paper.

## 1. Introduction

Many low-income countries (LICs) face the dual challenge of high rates of under-nutrition and increasing levels of overweight and obesity, resulting from a rise in obesogenic dietary patterns and, to a lesser extent, decreased physical activity levels [1,2,3,4,5]. A “low-income country”, defined by the authors, is any country that is classified by the World Bank as having a “low income” or “lower-middle income” economy [6]. High quality data on food and nutrient consumption are critical for a spectrum of programs and policies relating to nutrition, food systems, agriculture, and agricultural value chains. For instance, understanding factors such as the drivers of consumer food choice and their links to both agricultural production decisions and the food environment require individual-level dietary data. Dietary data are needed to establish relationships between diets and health outcomes, design and evaluate food fortification programs, monitor food safety, and track shifting consumption patterns. Age and sex-specific dietary data are also needed to develop population-appropriate food-based dietary guidelines and food labels.

Despite the existence of well-established methods for collecting individual-level dietary data, including 24-h dietary recalls (24HR), food frequency questionnaires, and weighed food records, large-scale collection of dietary data in LICs remains rare. Moreover, there is a dearth of country-specific, or even regional-specific, food composition data for many LICs. A commonly heard refrain is that there is great need for more dietary data in LICs, but the high costs, time burden, and technical complexity are barriers to dietary data collection. As a result, food and nutrition policies are often designed using less appropriate alternative data sources, such as national food balance sheets and household consumption and expenditure surveys (HCES), or, worse yet, are developed without benefit of any evidence base. While alternative data sources like HCES can sometimes provide proxy estimates of average individual consumption at household or national levels and have the benefit of being collected routinely for other purposes, these estimates do not provide age and sex disaggregated data, which are essential to the design of appropriate programs and policies in specific at risk population groups [7,8]. These alternative data sources are therefore best viewed as a complement, not a substitute, for individual-level dietary data.

The first objective of this discussion paper is to provide an overview of the major barriers and bottlenecks to scaling up individual-level dietary data availability, access, and use in LICs. The second objective of this discussion paper is to introduce readers to the investments in technologies, research infrastructure, and capacity strengthening that the International Dietary Data Expansion (INDDEX) Project is undertaking to improve this situation. Research infrastructure includes, amongst other things, data collection instruments, knowledge-containing resources such as e-platforms and data banks, data management protocols and knowledge transfer [9]. The third objective of this discussion paper is to highlight areas of global need that will require additional partnerships and resources.

## 2. The Challenge: Barriers That Impede the Collection and Use of Dietary Data in Low-Income Countries

Despite the importance of dietary data for a broad range of policy and research applications, there are few examples of nationally representative individual-level dietary surveys in LICs. An added challenge is the lack of a centralized structure to house existing survey data. Therefore, the data, whether from large or small sample dietary surveys, are often not broadly accessible for use by researchers, policy makers, and other stakeholders. Research conducted by Pisa et al. underscores these constraints [10]. The authors surveyed researchers from 23 countries across Africa to assess current use of dietary assessment methods at a continental level. Based on responses from researchers in 18 countries, the inventory reported 23 distinct 24HR surveys of varying sample sizes, all of which had been performed using a traditional pen and paper method. The authors also uncovered several key barriers to the increased collection and use of dietary data, such as the need for increased funding for dietary assessment studies, improved training, and investment in the research infrastructure required to scale up and standardize the data collection process.

The following sections discuss these critical barriers facing LICs related to cost and time, research infrastructure investments, and capacity with a focus on the 24HR method. Twenty-four hour dietary recalls are the focus as they represent a standardized approach that can be applied across countries, unlike food frequency questionnaires, which require significant adaptation and validation prior to implementation. The 24HR method is also presented because it is considered less time consuming and burdensome than the weighed food record method.

### 2.1. Time and Cost of Conducting 24-h Recall Surveys

Researchers in LICs typically use a pen and paper-based 24HR questionnaire to collect quantitative dietary data on individual intakes. The standard pen and paper method for conducting 24HR surveys is costly to implement, due to the many inefficiencies and bottlenecks described in this and subsequent sections of the narrative. The literature offers little by way of detailed financial requirements of 24HR studies in LICs, however, one study examined the costs associated with eight surveys conducted in Africa and South Asia and found the average cost of a two-round, 24HR survey of 480 households in Sub-Saharan Africa to be approximately $247 per household [11]. This estimate includes human resources (e.g., externally-based researchers, enumerators, supervisors, drivers, data entry operators and data analysts), materials (e.g., household measures, computers/laptops, measurement boards), supplies (e.g., phone cards, questionnaires), transportation (e.g., vehicles, travel), and overhead costs.

Preparation for a traditional dietary assessment survey is time consuming. It typically requires developing lists of commonly consumed foods, ensuring that a complete food composition database (also sometimes referred to as a ‘food composition table’) is available in order to convert data collected on foods and recipes into nutrient intakes, creating context-specific portion size estimation aids, developing standardized recipes for mixed dishes in order to avoid collecting ingredient details at the household level, and developing conversion factors for non-standard portion sizes to gram weight equivalents. Additional time and cost may be required to add foods or nutrients to the food composition database (FCDB) that are missing. This approach often requires lab analyses to obtain compositional information. A less accurate but lower cost method involves imputing missing values using FCDB data from other countries, which may not reflect the nutritional content of the locally consumed foods.

An interviewer-administered pen and paper 24HR interview typically takes between 30–45 min to conduct in low-, middle-, and high-income countries alike [12,13]. Yet, while 24HR surveys are somewhat time consuming to conduct, the time spent entering, cleaning, processing, and analyzing the dietary data is much more intensive than the data collection itself. When dietary surveys are conducted in low-income countries, the food items within the 24HR questionnaire’s food list are not always pre-coded to their corresponding item in the FCDB, which means that an added step is required after data entry to code all food items and ingredients. Dietary data collection applications in use in high-income countries, such as the Automated Self-Administered 24HR (ASA24) [14] and Intake24 [15], often contain food lists that are pre-coded to corresponding items in food composition databases, thus removing the need for post-interview coding. When coding occurs after data collection, as it normally does in dietary surveys conducted in LICs, all portions consumed must be converted to gram weight equivalents and all food items and mixed dishes must be matched to corresponding compositional data from the FCDB before the data can be analyzed in a statistical program. This step can be particularly challenging and time-consuming since items in many FCDBs in low-income countries are not clearly defined, which hinders efforts to appropriately and accurately match foods reported in consumption surveys.

### 2.2. Lack of Investment in Dietary Assessment Research Infrastructure

Many of the aforementioned time and cost inefficiencies of collecting and processing individual-level dietary data stem from the weak research infrastructure in LICs that, if strengthened, could greatly streamline the process of data collection and analysis. Data collection platforms exist to automate interviewer-administered data collection in low-literacy populations. An automated data collection platform, defined by the authors, refers to dietary assessment software that is either interviewer-administered or self-administered and that runs on a tablet, laptop, or desktop computer either by accessing the web or offline. These platforms replicate a range of dietary assessment methods including 24HR, food frequency questionnaires, and food records. Automation can significantly reduce time required through the survey cycle, particularly with regard to data entry, cleaning, and processing [16]; in addition, it is possible that some timesaving occurs during the data collection process, for example, by pre-specifying the portion size estimation techniques to be used. Furthermore, automated dietary data collection can simplify interviews, reduce cognitive burden on both the respondent and the interviewer [17], and reduce the risk of errors by standardizing the data collection process and including quality control measures such as range checks.

Research evaluating four different electronic data capture systems—two Android platforms (CommCare and ODK Collect), one for PALM and Windows OS (Pendragon), and a custom built application for Android (Mobile InterVA (MIVA))—used for mobile health projects in rural Malawi concluded that, in the long term, electronic data capture is likely to be cheaper than pen and paper methods. These cost savings accrue as capacities are developed and the hardware costs are amortized, especially when factoring in the high returns associated with the improved data quality [18]. Yet, nearly all existing electronic 24HR data collection platforms were designed for use in high-income countries [19,20]. Though a handful of automated data collection tools have been used to collect dietary data in resource constrained contexts, for example, in India and Zambia, these were designed for specific research purposes and settings only, and cannot be adapted to other geographic locations [21,22].

Adaptation to new settings would be facilitated by investments in dietary assessment research infrastructure that reflects the data needs of LICs. Presently, the inputs required for conducting a 24HR survey, such as lists of commonly consumed foods and mixed dishes, portion conversion factors, yield and retention factors, and food composition databases, are usually not publicly accessible, if they exist at all (‘publicly accessible’ is generally understood to mean accessible via the web) [23]. The absence of online and publicly accessible databases for these inputs in LICs means researchers must reproduce them for each new 24HR survey. The inefficiency of ‘reinventing the wheel’ with each survey is therefore a major contributor to the time and cost burden of dietary assessment, and a deterrent to conducting more frequent individual-level dietary surveys [24].

The limited investment in FCDBs is one of the largest gaps in the dietary assessment research infrastructure of LICs and is cited as a major impediment to analysis and use of individual-level dietary data in this region [10,25]. When national FCDBs do exist, they frequently provide only partial information on critical foods and nutrients, unclear definitions of key nutrients such as vitamin A and, often, inadequate documentation, which precludes any assessment of data quality. Many studies rely on outdated food composition data or resort to using an amalgamation of sources borrowed from high-income countries, such as from the United States Department of Agriculture’s National Nutrient Database for Standard Reference [26]. Borrowed data are often from high-income countries’ FCDBs published in the 1960s to 1980s, which have since been updated with higher quality data in the original countries. A recent analysis of animal-source food data from select FCDBs across Sub-Saharan Africa illustrates this point [25]. The authors found that borrowed values comprised as much as 88% of all animal-source food entries in Lesotho, 85% in The Gambia, and 69% in Uganda. The tendency to borrow data from other FCDBs is unsurprising considering the relatively high costs associated with conducting laboratory analyses of local foods as well as the scarcity of high-quality chemical laboratories in many LICs. Countries that do have their own FCDBs rarely update them due to resource constraints and the lack of required specialized skills [10]. This is increasingly an issue as food supplies have changed rapidly in the past 30 years [4], particularly with the proliferation of ultra-processed food products (ultra-processed food products are intended to distinguish between unprocessed or minimally processed foods and processed culinary or food industry ingredients—ultra-processed food products are a combination of the other two in order to create convenient, and palatable ready to eat, with examples including biscuits, cakes, ice cream, hot dogs, sausages, etc. [27]). Many of these foods have inaccurate and incomplete nutrient composition information on the label, if they contain a label at all [28,29,30,31]. Food labeling in LICs is also constrained by the scarcity of individual level dietary data, particularly population-specific portion size information, which is often used as a reference when developing the serving sizes of foods listed on the product’s nutrition label. In most cases these food products are not listed in available FCDBs in LICs [23,32]. Therefore, foods within FCDBs that were constructed in the 1970s and 1980s may reflect only a portion of the foods in a country’s food system with associated inadequate compositional data.

Furthermore, the genetic, soil, and agro-ecological differences across geographic regions can affect the nutrient profile of the food, so that food grown in one geographic area may have different mineral and vitamin levels than the same foods grown in other geographic areas [23,32,33]. For example, nutrient values of commercially raised breeds of animals, which are used as the reference value in high-income countries, should not be compared to animals raised in LICs using indigenous breeds of livestock. In many LICs, the feed given to livestock may be both unstandardized and unfortified, and livestock are often raised in pastoral systems that provide different sources of animal feed that vary in nutrient composition [25,34]. Similarly, nutrient levels can vary substantially between different varieties of the same food. For instance, depending on the variety, the protein content of rice can range from 5.6 to 14.6 g (per 100 g edible portion, raw), while micronutrient contents can vary by a magnitude of two or three [35]. Nutrient contents of wild or biodiverse colorful varieties tend to be higher than the nutrient contents of less colorful varieties produced using mainstream agricultural practices. Therefore, FCDBs that rely on data borrowed from high-income countries or regions may significantly underestimate (and sometimes overestimate) the true nutrient contents of the foods in the local food supply. Variety-specific differences can represent the difference between nutrient deficiencies and nutrient adequacy in populations and individuals [36].

In addition, it is expected that climatic shifts will further affect levels of key nutrients by the mid-century. Recent research has demonstrated that the impact of elevated levels of atmospheric carbon dioxide (CO2) expected by 2050 will result in a reduction of zinc and iron concentrations in wheat [37]. The effect of climatic shifts on nutrient contents of foods is yet another concern that will have implications for the accuracy of nutrient estimates that rely on current FCDBs.

This array of issues highlights the need for increased investment in FCDBs to ensure that they are up-to-date and reflect the true nutrient content of locally consumed foods. Factors that introduce variation in composition within a single geography (e.g., seasonal differences in growing conditions, soil composition) should be taken into account when FCDBs are prepared. Accurate nutrient composition data are critical to nutrition scientists, food producers, food processors, retailers, consumers, and government agencies. There is also growing interest in nutrient composition data from a broader group of stakeholders, including economists who use FCDBs to estimate the caloric content of foods reported in HCES for poverty estimation and a specialized set of engineers, computer scientists, and healthcare workers who are developing automated data collection platforms that are seamlessly linked with FCDBs.

### 2.3. Lack of Centralized Dietary Data Dissemination Platforms

The aforementioned bottlenecks relate primarily to the collection and analysis of new dietary data, however, there are also impediments to accessing and using existing dietary survey results. At present there is no publicly available, centralized repository of individual-level dietary survey data that is analogous to the Food and Agriculture Organization of the United Nations (FAO) FAOSTAT platform for accessing food balance data [38] or the International Household Survey Network, where HCES are often housed [39]. This issue has been highlighted recently in two notable publications, a report on “Food systems and diets: Facing the challenges of the 21st century” published by the Global Panel on Agriculture and Food Systems for Nutrition [40]; and a paper by Haddad, Hawkes et al. [41] published in *Nature* which lists as one of the ten global research priorities for food the need to “make more data on diets widely available”. Therefore, an important complement to any investment in global research infrastructure should also include investments in online databases to house and freely share individual-level dietary data to ensure wider access and use. An “open data” model is a critical step towards dietary data being more readily accessible and used for various purposes including developing population-appropriate food-based dietary guidelines and food labels, designing and evaluating food fortification programs, and monitoring food safety.

### 2.4. Low Capacity and Limited Experience Linking Food Consumption Data with Policy

The lack of targeted investment in capacity development and dietary assessment skills in LICs remains a major constraint. Without adequate skills and the appropriate enabling environment at a country level, researchers may not know how to conduct individual-level dietary surveys or leverage the results for maximum policy impact. For example, limited experience collecting and analyzing individual-level dietary data may hinder the ability of country governments to draft their own food-based dietary guidelines, an important step in operationalizing and aligning national food policies for healthy diets.

A recent systematic assessment of nutrition capacity at the individual level (tools and skills), the organizational level (staff and infrastructure), and the systemic level (governance, coordination mechanisms, and information management) from 13 West African countries found that there was a critical shortage of trained nutrition professionals in the region [42]. In Burkina Faso, for instance, the authors noted low capacity to produce nutrition graduates: 15 nutrition graduates are produced annually, which the authors deemed insufficient to adequately address the nutrition needs of the country. Of equal concern is the fact that, none of the West African countries surveyed had a well-defined nutrition information system to inform decision-making processes [42]. While these results are specific to West Africa, they exemplify the paucity of investment in nutrition information capacity faced in many countries.

An issue closely related to that of capacity and nutrition information systems is how best to leverage dietary data to influence decision-making. Insufficient attention has been given to presenting and communicating results from dietary surveys in order to influence policy decisions. As discussed previously, a necessary complement is ensuring that microdata and dietary indicators are publicly available and freely accessible for use in decision-making processes.

## 3. An Integrated Solution: Innovative Tools to Alleviate Bottlenecks and Scale up Dietary Assessment Research in Low-Income Countries

The INDDEX Project endeavors to alleviate many of the aforementioned bottlenecks through investment in a comprehensive dietary assessment platform that is targeted to the needs of stakeholders in LICs. The platform, called INDDEX24, will consist of a mobile application for the collection of individual-level dietary data using the 24HR method (referred to as the *dietary recall mobile app*) that will be linked to a web database application (referred to as the *web database*) that will house country and region-specific inputs required to collect and process individual-level dietary data. The following sections describe the systematic process used to determine the INDDEX24 technical specifications and provide an overview of the dietary recall mobile app and web database that will comprise the INDDEX24 platform. A final section presents other initiatives supported through INDDEX that aim to broaden dietary data availability, access, and use in LICs, namely, investments in FCDBs, the FAO/World Health Organization Global Individual Food consumption data Tool (FAO/WHO GIFT), and capacity development.

### 3.1. A Solution to the High Time and Cost of Conducting 24-h Recall Surveys in LICs: INDDEX24 Mobile Application for Dietary Data Collection

#### 3.1.1. Conceptualization and Development Process

A multipronged approach was used to develop INDDEX24’s technical specifications. First, a dietary assessment expert with in-depth knowledge and experience conducting dietary surveys in low-income countries was hired to draft an initial list of priority technical specifications for a dietary recall mobile app. Priority specifications for the INDDEX24 mobile app included the use of the multiple pass approach, contextual adaptability, availability to users at low or no cost, capability of offline data collection and interviewer-administration, use of open source code, and user friendliness (for a complete list and description of the priority technical specifications see [19]). The multiple pass method has been shown to prompt respondents’ memory of foods consumed in increasing detail during each successive pass [43]. Many existing 24HR platforms, such as Intake24 and ASA24, are also modeled on the multiple pass method [14,15]. The authors defined contextually adaptable as allowing researchers to upload population-specific data inputs such as food lists (lists of most commonly consumed single item foods), recipe lists (lists of most commonly consumed multi-ingredient dishes, broken down into information on individual ingredient quantities, with details provided on cooking methods and the final weight of the cooked dish), yield and nutrient retention factors, FCDBs, and lists of portion conversion factors, thus enabling the tool to be used in a range of settings. This feature of contextual adaptability refers to data collection platforms in particular. A number of dietary processing platforms have been developed that permit users to upload context specific databases for processing of dietary intake data (e.g., CSDietary, Lucille, and World Food Dietary Assessment System); however, these tools are not designed for data collection. The flexibility for adaptation combined with the specification that the data collection platform be accessible to researchers at no or low cost were deemed necessary criteria to reduce the current high cost of conducting dietary assessment surveys and to facilitate the scale-up of dietary data collection in LICs.

Administration of the tool by a literate interviewer was necessitated by the low respondent literacy rates that exist in many LICs. According to the Central Intelligence Agency World Factbook, there are approximately 781 million illiterate adults (>15 years) globally and more than three-quarters of them live in South Asia, West Asia, and sub-Saharan Africa, with women comprising almost two-thirds of all of the illiterate adults in the world [44]. The need for offline data collection was deemed essential for LICs where reliable Internet access is lacking, particularly in rural areas. Indeed, many LICs are affected by the ‘global digital divide’, a term used to explain the uneven provision and development of Internet services internationally, with the African continent lagging far behind Asia and the Americas [45]. The authors wanted to ensure that the source code of the dietary recall mobile app would be a global public good and therefore use of open source code was deemed an essential specification. Finally, stakeholders concurred that a dietary platform designed for LICs must be as straightforward as possible to use given that many LICs have limited experience conducting dietary assessment surveys.

The second step in defining INDDEX24’s technical specifications involved conducting a systematic literature review of existing computer-based 24HRs in conjunction with key-informant interviews with representatives of institutions hosting these platforms [19]. The objective of the review was to assess the landscape of available 24HR platforms and determine their suitability for use in LIC contexts. The systematic literature review conducted by the authors as well as a more recently conducted review by Timon et al. [20] identified a number of computer-based and web-based 24HR platforms that have been developed for dietary assessment [14,15,19,21,46,47,48,49,50,51,52,53,54,55,56,57,58,59,60,61]. As mentioned previously, the majority of 24HR platforms have been developed for use in high-income countries in North America and Europe. Ultimately, the authors determined that no existing 24HR platform met the priority technical specifications for use in a LIC context specified above. The authors thus decided that the INDDEX Project should further refine its initial technical specifications and proceed with the development of its own 24HR platform, INDDEX24.

The third step in refining the initial set of INDDEX24’s technical specifications included an evaluation of the specifications in terms of their costs, benefits, and validity in the context of LICs by members of the INDDEX Project’s Technical Advisory Group. Fourth, the INDDEX Project sought input on the feasibility of the technical specifications from external information technology experts. Formal development of the INDDEX24 platform began in early 2017. To ensure that INDDEX24 is maximally useful and user friendly for stakeholders in LICs, input into the platform’s technical specifications will be solicited from potential end-users throughout the development process, and the tools will be refined accordingly.

#### 3.1.2. Functionality of INDDEX24 Mobile Application for Dietary Data Collection

The INDDEX24 dietary recall mobile app will be built using Commcare, an open source mobile data collection platform designed for use in low-resource settings [62]. To use the mobile app, researchers will log onto a cloud-hosted researcher website, where they will be able to access or upload the types of population-specific data inputs mentioned above that will facilitate processing of 24HR dietary data collected using the mobile app. These inputs will be housed in the web database, which is described below. Additional anticipated features of the researcher website include the ability to:

- Administer project user accounts (e.g., accounts for enumerators)
- Monitor survey data collection progress
- Make content tweaks to the application’s questionnaire using an application builder
- Download full and de-identified data sets for analysis

It is expected that all food data within INDDEX24 will be coded using the FoodEx2 coding system, a standardized system for classifying and describing food that has been developed by the European Food Safety Authority (EFSA), and enhanced in 2015 through a collaboration between EFSA and FAO, to cover foods at the global level [63]. Food items for the food and recipe lists will be drawn from the country’s FCDB or local surveys and coded accordingly using FoodEx2, thereby facilitating the matching of reported foods and mixed ingredient dishes with corresponding food composition information. In most cases, standard recipes will serve to break down the recipe into its ingredients, which will then be used to estimate the food or nutrient intakes. The dietary recall mobile app will allow for the collection of household recipe information if a respondent reports consuming a mixed dish that does not correspond to any standardized recipes listed within the app.

Following a review by INDDEX team members of innovative, high tech methods for portion size estimation, such as automated food imaging, investigators determined that none of these cutting-edge options is currently ready for large-scale surveys, particularly in LIC contexts. The dietary recall mobile app will therefore draw on portion size estimation methods commonly used in LICs, such as direct weighing of either the actual foods if they are available in the household or salted replicas if they are not, household measures in combination with calibrated measuring containers, and possibly images of graduated food portions. Although atlases of food images represent the most commonly used portion size estimation technique in existing 24HR platforms [20], this approach is less commonly used in LICs. The INDDEX Project is collaborating with researchers at RTI International to assess the validity and accuracy of food images as portion size estimation aids in LICs. The results from this study will be used to determine if the dietary recall mobile app should include calibrated food images as an option for portion size recall. The dietary recall mobile app is expected to be able to convert food portions reported in non-standard units into gram-weight equivalents by linking to context-specific portion size conversion factors that will be housed within the web database.

The dietary recall mobile app will contain a number of quality control features. These features include controls to prevent entry of implausible values, pre-definition of the portion size estimation methods depending on the type of food reported, definition of a standardized approach for use when the respondent does not know the quantity consumed, and real-time calculation of the respondent’s reported energy intake in order to identify possible under- and over-reported intakes [64].

### 3.2. A Solution to the Limited Investment in Dietary Assessment Research Infrastructure in LICs: INDDEX24 Web Database for Dietary Data Processing

The INDDEX24 web database will provide centralized access to the various data inputs needed to collect and process dietary data using the dietary recall mobile application, in a standardized format that can be linked to the dietary data collection app. The web database’s default user permissions will allow users to select their country or region of interest and obtain the corresponding country or region-specific data necessary for using the dietary recall mobile application. Although the initial version of the web database will include data for only a small number of countries, the number of countries represented in the database and its overall comprehensiveness is expected to increase over time as researchers upload and share their data for their own use and for access by others.

Appropriate administrative structures will be implemented to enable users to add their own information to the existing database while maintaining quality controls. The web database’s administrative protocols will be modeled on existing guidelines for ensuring data quality when borrowing compositional data [65,66]. In all cases, databases will follow a pre-defined structure and format, which is necessary to ensure compatibility with the dietary recall mobile app, but also promotes standardization in dietary data collection, processing, and management.

The INDDEX24 dietary recall mobile app and web database will be integrated with one another, which will enable users to download the required country-specific data inputs to the dietary recall mobile app for use during data collection. Integration will also ensure that any updates made to the web database will be readily available to users of the dietary recall mobile app.

At the end of the project, an ideal host envisaged for the web database would be an international organization such as FAO, considering its mandate to “collect, analyze, interpret, and disseminate information relating to nutrition, food and agriculture” (Article 1 of the FAO constitution) [67]. The web database would provide a useful complement to the information on food consumption that is currently available on FAOSTAT. However, arrangements still need to be made and resources for the maintenance of such a database still need to be identified in order to ensure the sustainability of the platform.

Relatedly, the INDDEX Project is investing in updates to the West African Food Composition Table (WAFCT) [68]. The updated WAFCT, which is expected to be publicly available in 2018, will include more analytical data than the 2012 version, more foods, and approximately 100 recipes, as well as food composition information on fermented foods. It is recognized that more FCDBs should be updated and be included in INDDEX24, which would increase significantly the value of both tools. The INDDEX Project is not able to support comprehensive updates to multiple FCDBs, nor is it able to significantly invest in global human resource and technical capacity related to FCDB quality and use. These are important endeavors that can be realized with additional resources.

The INDDEX24 web database will represent the first publicly accessible central repository of global data inputs required for conducting 24HR survey research in LICs. While other organizations provide researchers with assistance in locating global food composition information (e.g., the FAO International Network of Food Data Systems (INFOODS) and the recently released International Life Sciences Institute (ILSI) Research Foundation Catalogue of Food Composition Databases and Tables (CatFCDB) [69], which offers contact information for connecting with FCDB databased managers), the actual content of the FCDBs cannot be accessed directly through these sites. By centralizing the location of FCDB, recipe, and portion conversion data, INDDEX24’s web database aims to circumvent many of the steps typically required to prepare for a 24HR survey in order to increase the overall efficiency of the research process.

#### Evaluation of INDDEX24: Feasibility, Validation, and Cost Studies

The development of both the dietary recall mobile app and web database will utilize an iterative approach, which will involve frequent tests and refinements until the products function according to envisioned technical specifications. Once a prototype is ready, the INDDEX Project plans to conduct a feasibility study of the dietary recall mobile app and integrated web database in Bangladesh and Burkina Faso. The protocols for these studies will integrate lessons learned from feasibility and validation studies conducted in upper income countries using existing 24HR platforms [70]. The study will assess the ease of use, including the feasibility of uploading country-specific databases from the web database onto the mobile app and the usability of the mobile app’s interface. Based on the results of feasibility testing, the dietary recall mobile app and web database will be refined. Following refinement, the INDDEX Project intends to conduct a validation study of the dietary recall mobile app by testing it against the doubly labeled water method and possibly weighed food records. A complementary study on the costs associated with using the mobile tools relative to the traditional pen-and-paper method will also be conducted. A first version of the dietary recall mobile app and web database is expected to be publicly available by the end of the INDDEX Project.

### 3.3. A solution to the Low Use of Food Consumption Data for Policy-Making in LICs: FAO/WHO GIFT and Capacity Development Activities

The INDDEX Project is also investing in the development of the FAO/WHO Global Individual Food consumption data Tool (FAO/WHO GIFT) in order to increase public access to dietary indicators in useful and usable formats. FAO/WHO GIFT will collect, harmonize and disseminate 24HR and food record data available at national and sub-national levels all over the world through a FAO hosted web-platform [71]. The development of this platform is done in synergy with a complementary initiative at Tufts University, the Global Dietary Database (GDD) [72], which collates both individual and household food consumption data. The GDD platform currently provides age and sex disaggregated food consumption data in relation to 14 dietary risk factors (including eight food groups) from all geographical areas of the world in order to assess the contribution of dietary risk factors to the global burden of disease. A unique feature of the FAO/WHO GIFT platform is that it will disseminate datasets at a very high level of disaggregation (i.e., microdata on individual consumption at the level of food items) after a recoding process with the FoodEx2 description and categorization system. FAO/WHO GIFT is intended for use by both experts who may want to analyze microdata and by laypeople with low scientific literacy. FAO/WHO GIFT will provide food-based nutrition indicators in the form of infographics so that policymakers in low and middle income countries will be better able to make evidence-based policy decisions concerning food systems, nutrition, and agriculture. The INDDEX Project’s intention is to design INDDEX24 so that dietary data, collected using its dietary recall mobile app, can be easily uploaded into FAO/WHO GIFT where they can be accessed and used for policy relevant purposes.

Stakeholders in Bangladesh and Burkina Faso will be involved throughout the technology and infrastructure development process not only to ensure that the tools developed by the INDDEX Project respond to the needs of end users in LICs, but to develop capacity to scale up dietary data collection, analysis, and use within these countries. Once completed, the INDDEX Project will conduct in-country trainings and demonstration analyses of the INDDEX24 platform. The INDDEX Project also intends to focus on capacity development in additional LICs through the use of virtual regional working groups. The INDDEX Project is not able make significant investments in workforce development in nutrition and dietary assessment. A concerted global effort is therefore necessary to sustain capacity investments in dietary assessment in LICs.

## 4. Conclusions

INDDEX24 is expected to produce a transformative contribution to the landscape of dietary assessment in LICs. Though there are examples of 24HR data collection applications that have been applied in LICs, these programs were designed for specific contexts or with proprietary code, which hinders adaptability and open access. As such, INDDEX24 will represent, to our knowledge, the first dietary assessment platform that is intended for flexible adaptation to different contexts. It is anticipated that investments in the research infrastructure, the introduction of a publicly accessible platform for dietary data collection and processing, and complementary capacity development initiatives will significantly reduce existing bottlenecks that inhibit the collection and use of individual-level dietary data in LICs. These contributions will enable stakeholders in LICs to design evidence-based programs and policies to address current and emerging diet-related policy challenges. In order to continue to strengthen global dietary data infrastructure, particularly in the realm of food composition data and human resources for dietary assessment, additional broad international commitment and investment will be needed.
